# Supplementary material for: A Critical View of the Application of the APEX Software (Aqueous Photochemistry of Environmentally-Occurring Xenobiotics) to Predict Photoreaction Kinetics in Surface Freshwaters
Source: Molecules. 2019 Dec 18;25(1):9. doi: 10.3390/molecules25010009 (PMC7017383; doi:10.3390/molecules25010009)
Supplement: Supplementary file 1 [file molecules-25-00009-s001.zip › 3.2.4_gcc-4.4.0.zip!/3.2.4_gcc-4.4.0/3.2.4_gcc-4.4.0/doc/octave/html/interpreter/Character-Class-Functions.html]

Character Class Functions - Untitled


Previous: String Conversions,
Up: Strings


---

### 5.7 Character Class Functions

Octave also provides the following character class test functions
patterned after the functions in the standard C library. They all
operate on string arrays and return matrices of zeros and ones.
Elements that are nonzero indicate that the condition was true for the
corresponding character in the string array. For example,

```
     isalpha ("!Q@WERT^Y&")
          => [ 0, 1, 0, 1, 1, 1, 1, 0, 1, 0 ]
```

— Mapping Function: **isalnum** (s)  
> Return 1 for characters that are letters or digits (`isalpha
> (`s`)` or `isdigit (`s`)` is true).

— Mapping Function: **isalpha** (s)  
— Mapping Function: **isletter** (s)  
> Return true for characters that are letters (`isupper (`s`)`
> or `islower (`s`)` is true).

— Mapping Function: **isascii** (s)  
> Return 1 for characters that are ASCII (in the range 0 to 127 decimal).

— Mapping Function: **iscntrl** (s)  
> Return 1 for control characters.

— Mapping Function: **isdigit** (s)  
> Return 1 for characters that are decimal digits.

— Mapping Function: **isgraph** (s)  
> Return 1 for printable characters (but not the space character).

— Function File: **isletter** (s)  
> Returns true if s is a letter, false otherwise.
>
>
>
> **See also:** isalpha.

— Mapping Function: **islower** (s)  
> Return 1 for characters that are lower case letters.

— Mapping Function: **isprint** (s)  
> Return 1 for printable characters (including the space character).

— Mapping Function: **ispunct** (s)  
> Return 1 for punctuation characters.

— Mapping Function: **isspace** (s)  
> Return 1 for whitespace characters (space, formfeed, newline,
> carriage return, tab, and vertical tab).

— Mapping Function: **isupper** (s)  
> Return 1 for upper case letters.

— Mapping Function: **isxdigit** (s)  
> Return 1 for characters that are hexadecimal digits.

— Function File: **isstrprop** (str, pred)  
> Test character string properties. For example,
>
> ```
>           isstrprop ("abc123", "alpha")
>           => [1, 1, 1, 0, 0, 0]
> ```
>
> If str is a cell array, `isstrpop` is applied recursively
> to each element of the cell array.
>
> Numeric arrays are converted to character strings.
>
> The second argument pred may be one of
>
> `"alpha"`: True for characters that are alphabetic `"alnum"` `"alphanum"`: True for characters that are alphabetic or digits. `"ascii"`: True for characters that are in the range of ASCII encoding. `"cntrl"`: True for control characters. `"digit"`: True for decimal digits. `"graph"` `"graphic"`: True for printing characters except space. `"lower"`: True for lower-case letters. `"print"`: True for printing characters including space. `"punct"`: True for printing characters except space or letter or digit. `"space"` `"wspace"`: True for whitespace characters (space, formfeed, newline, carriage return, tab, vertical tab). `"upper"`: True for upper-case letters. `"xdigit"`: True for hexadecimal digits.
>
>
>
>
> **See also:** isalnum, isalpha, isascii, iscntrl, isdigit, isgraph, islower, isprint, ispunct, isspace, isupper, isxdigit.
